# An Enhanced Belief Propagation Flipping Decoder for Polar Codes with Stepping Strategy

**DOI:** 10.3390/e24081073

**Published:** 2022-08-03

**Authors:** Xiaojun Zhang, Yimeng Liu, Chengguan Chen, Hua Guo, Qingtian Zeng

**Affiliations:** 1 College of Electronic and Information Engineering, Shandong University of Science and Technology, Qingdao 266590, China; 202082130008@sdust.edu.cn (Y.L.); 201982130005@sdust.edu.cn (C.C.); stonestrong@sdust.edu.cn (H.G.); qtzeng@sdust.edu.cn (Q.Z.); 2 State Key Laboratory of High-End Server and Storage Technology, Jinan 250101, China

**Keywords:** polar code, belief propagation, bit-flipping, stepping

## Abstract

The Belief Propagation (BP) algorithm has the advantages of high-speed decoding and low latency. To improve the block error rate (BLER) performance of the BP-based algorithm, the BP flipping algorithm was proposed. However, the BP flipping algorithm attempts numerous useless flippings for improving the BLER performance. To reduce the number of decoding attempts needed without any loss of BLER performance, in this paper a metric is presented to evaluate the likelihood that the bits would correct the BP flipping decoding. Based on this, a BP-Step-Flipping (BPSF) algorithm is proposed which only traces the unreliable bits in the flip set (FS) to flip and skips over the reliable ones. In addition, a threshold β is applied when the magnitude of the log–likelihood ratio (LLR) is small, and an enhanced BPSF (EBPSF) algorithm is presented to lower the BLER. With the same FS, the proposed algorithm can reduce the average number of iterations efficiently. Numerical results show the average number of iterations for EBPSF-1 decreases by 77.5% when *N* = 256, compared with the BP bit-flip-1 (BPF-1) algorithm at $E_{b}/N_{0}$ = 1.5 dB.

## 1. Introduction

Polar code is the first error-correcting code [1] which has achieved the Shannon limit. It has been adopted as the fifth generation (5G) wireless communications standard [2]. Not only does it have a strong error correction capability, but its encoding and decoding complexity is also affordable compared with the Low Density Parity Check (LDPC) [3] and Turbo codes [4].

The Successive Cancellation (SC) algorithm, which was proposed by Arıkan [5], is one of the most common decoding methods for polar codes and has attracted widespread attention. The original SC algorithm has been optimized by the relevant scholars, producing optimizations such as the SC list (SCL) [6], SC flip (SCF) [7] and dynamic SC flipping (DSCF) [8] algorithms. The Cyclic redundancy check (CRC)-aided SC list (CA-SCL) [9] decoder was introduced to improve the BLER of polar codes and has become a baseline algorithm used in the standardization process. Compared with the SC and its other optimized algorithms, the Belief Propagation (BP) algorithm [10] with parallel decoding has great advantages in terms of its throughput and decoding latency. In addition, BP decoding is expected to support polar codes of 5G standard in practical applications with a set of hardware units. However, the BP decoder will not terminate until the maximum number, the termination scheme suffers from lack of flexibility, and introduces great computational complexity. Moreover, the BLER performance of BP is uncompetitive.

To improve the original BP decoding performance, many methods to improve the BP-based algorithm have been proposed—for example, the BP list (BPL) [11] decoder. When using the standard polar code decoding factor graph (DFG), the original BP algorithm may generate incorrect decoding results because the transmission process of the messages is modified. However, the BPL decoder uses the permuted version of the standard graph and can produce the correct estimation result. A permuted factor graph is introduced in [12,13] based on the BPL decoder. The guessing algorithm [14] was first proposed in the LDPC codes and then introduced to the BP decoding, which guesses the oscillating bits by assigning the priority log-likelihood ratios (LLRs), the maximum LLR magnitude, and repeating the original BP decoding to achieve good decoding results. The BP Bit-Flip (BPF) [15] algorithm, which is different from other BP-based evolutionary algorithms, utilizes the information in the decoding process to make it more targeted to the codeword being decoded.

BP flipping algorithms identify and then flip error-prone positions to improve decoding performance. For polar codes, bit-flipping strategies have been applied to both SC and BP decoding. However, the principles of flipping strategies on SC and BP decoding are different due to their different decoding schedules. The key to the BP flipping algorithm is to construct a flip set (FS), which consists of the error-prone bits. FS can accurately indicate the error bits, thus flipping the bits within FS can narrow the search space. Initially, the FS was constructed in SC [7], which proposed a progressive multi-bit-flipping algorithm. Under the Gaussian approximation (GA) construction, it was generated according to the Rate-1 nodes called the critical set (CS). The bits identified in the CS may exhibit a high possibility of error in the BP decoding. Thus, the CS is introduced to BP and the constructed CS with order *T* (CS-*T*) [15], where *T* denotes the size of the CS. By analyzing the behavior of the incorrect decoding results of the bit-flipping BP decoder with CS, a BP decoder with multiple bit-flipping sets (BFSs) and stopping criteria (BP-MF-MC) was proposed [16]. The CS is relatively static and the multiple BPSs have more complexity, so the Generalized BP Bit-flipping (GBPF) Decoder [17] with a redefinition of BP bit-flipping was proposed. The error-prone bits in the FS consist of the unfrozen bits with small LLR magnitudes from the outputs, which contribute to a better performance. Subsequently, the GBPF decoding was extended to a higher-order GBPF algorithm (GBPF-Ω) [18], where the maximum bit-flipping order is Ω. To narrow the BLER performance gap between BP-based decoders and CA-SCL decoder, the bit-flip method was also introduced into the BPL decoder and the noise-aided BPL (NA-BPL) decoder [19] was proposed. Above all, although flipping error-prone positions after the failure of the first decoding trail will improve the performance of BP-based decoder, the characteristics of the bits in FS are selected to be flipped in turn and many of them have no contribution to correcting the error frame, which will result in a huge amount of invalid repeated decoding attempts, giving rise to more latency.

This paper aims to reduce the number of decoding attempts in the BP flipping algorithms. The main contributions are summarized as follows:

- A stepping strategy is proposed. We first analyze the behavior of FS and find that only a few bits in the FS could correct the error frame. Therefore, a concept to evaluate the likelihood of bits correcting the trajectory of BP decoding is presented to judge whether the bits in FS should be flipped or not. The judgement condition determines whether flipping the bits in the FS is helpful in correcting error frames.
- Based on the stepping strategy, an optimization algorithm for the BP flipping algorithm, the BP step-flipping (BPSF) algorithm, is proposed. The algorithm flips only unreliable bits in FS and steps reliable bits to shrink the number of flipping attempts necessary. Similarly, the stepping strategy is also added into the GBPF-Ω algorithm [17,18] to reduce the number of flipping attempts.
- In addition, we notice that some effective flipping bits may be skipped over when the LLR magnitude is small. We further propose the enhanced BPSF-Ω (EBPSF-Ω) algorithm, which adopts a threshold to identify the unreliable bits and lowers the block error rate (BLER). The numerical results obtained indicate that the average number of iterations can be significantly reduced for the EBPSF-1 and EBPSF-2 algorithms at the low $E_{b}/N_{0}$, compared with the BPF-1 and BPF-2 flipping algorithms when the code length is 256.

The remainder of this paper is organized as follows. Section 2 reviews the polar code, the original BP algorithm, and the BP flipping algorithm. The BPSF-Ω algorithm with a threshold is proposed in Section 3. Section 4 analyzes the decoding performance. Conclusions are drawn in Section 5.

## 2. Preliminary

In this paper, we use calligraphic characters, such as R, to denote sets. We write *r*, r, and R to denote a scalar, a vector, and a matrix, respectively. In this section, we first describe the polar codes. Then, we briefly introduce the original BP algorithm. Finally, the BP flipping algorithm is presented.

### 2.1. Polar Code

After channel combining and channel splitting, *N* independent copies of binary-input discrete memoryless channels are converted to *N* split channels with different capacities [18]. Some of these have a high channel capacity, which means that the channel is more reliable for transmitting information, and some of them have a low capacity. Polar codes use high-capacity channels to transmit information bits and CRC bits, and the rest of the channels are used to transmit frozen bits. In this paper, the frozen bits are fixed to zero.

Polar code can be represented as *P* (*N*, *K*), where *N* is the code length of the polar code and *K* represents the length of the information bits. Meanwhile, (N−K) represents the length of frozen bits. The code rate is *R* = K/N. The *K* information bits comprise (K−r)-bit data and *r*-bit CRC. The set of information bits and frozen bits are denoted as A and $A^{C}$, respectively. The encoding process can be expressed as

(1) $$\begin{array}{c} x_{1}^{N}=u_{1}^{N}G_{N}, \end{array}$$

where $x_{1}^{N}$ = $\left\{x_{1},x_{2},\ldots ,x_{N}\right\}$ represents the codeword and $u_{1}^{N}$ = $\left\{u_{1},u_{2},\ldots ,u_{N}\right\}$ denotes the source vector which is mixed with the information bits $u_{A}$ and the frozen bits $u_{A^{C}}$. The generator matrix is represented as $G_{N}$=$B_{N}F^{⊗n}$, where $B_{N}$ is the bit-reversal permutation matrix [20], and $F^{⊗n}$ denotes the *n*-th Kronecker power of $n={log}_{2}N$ and

(2) $$F=\left[\begin{array}{cc} 1 & 0\\ 1 & 1 \end{array}\right].$$

The BP decoding is initiated from the received value $y_{1}^{N}$=$\left\{y_{1},y_{2},\ldots ,y_{N}\right\}$. The decoder generates an estimation ${\hat{u}}_{i}$ of $u_{i}$ based on the received $y_{1}^{N}$ as

(3) $$\begin{array}{c} {\hat{u}}_{i}=\left\{\begin{array}{c} 0,\;\mathrm{if}\;i\in A^{C},\\ 0,\;\mathrm{if}\;i\in A\;\mathrm{and}\;\frac{W_{N}^{\left(i\right)}\left(y_{1}^{N},{\hat{u}}_{1}^{i-1}\left|u_{i}=0\right.\right)}{W_{N}^{\left(i\right)}\left(y_{1}^{N},{\hat{u}}_{1}^{i-1}\left|u_{i}=1\right.\right)}\geq 1,\\ 1,\;\mathrm{if}\;i\in A\;\mathrm{and}\;\frac{W_{N}^{\left(i\right)}\left(y_{1}^{N},{\hat{u}}_{1}^{i-1}\left|u_{i}=0\right.\right)}{W_{N}^{\left(i\right)}\left(y_{1}^{N},{\hat{u}}_{1}^{i-1}\left|u_{i}=1\right.\right)}<1. \end{array}\right. \end{array}$$

A binary input memoryless channel $W_{n}$ generates *N* sub-channels by channel splitting, defining $W_{n}^{\left(i\right)}\left(y_{1}^{N},\left.{\hat{u}}_{1}^{i-1}\right|u_{i}\right)$, *i* = *1*, *2*, …, *N*. Let the LLR of $W_{n}^{\left(i\right)}\left(y_{1}^{N},\left.{\hat{u}}_{1}^{i-1}\right|u_{i}\right)$ be defined as

(4) $$\begin{array}{c} L_{n}^{\left(i\right)}\left(y_{1}^{N},{\hat{u}}_{1}^{i-1}\right)=\ln\left(\frac{W_{n}^{\left(i\right)}\left(y_{1}^{N},{\hat{u}}_{1}^{i-1}|u_{i}=0\right)}{W_{n}^{\left(i\right)}\left(y_{1}^{N},{\hat{u}}_{1}^{i-1}|u_{i}=1\right)}\right). \end{array}$$

### 2.2. Original BP Decoding Algorithm

**Figure 1:**
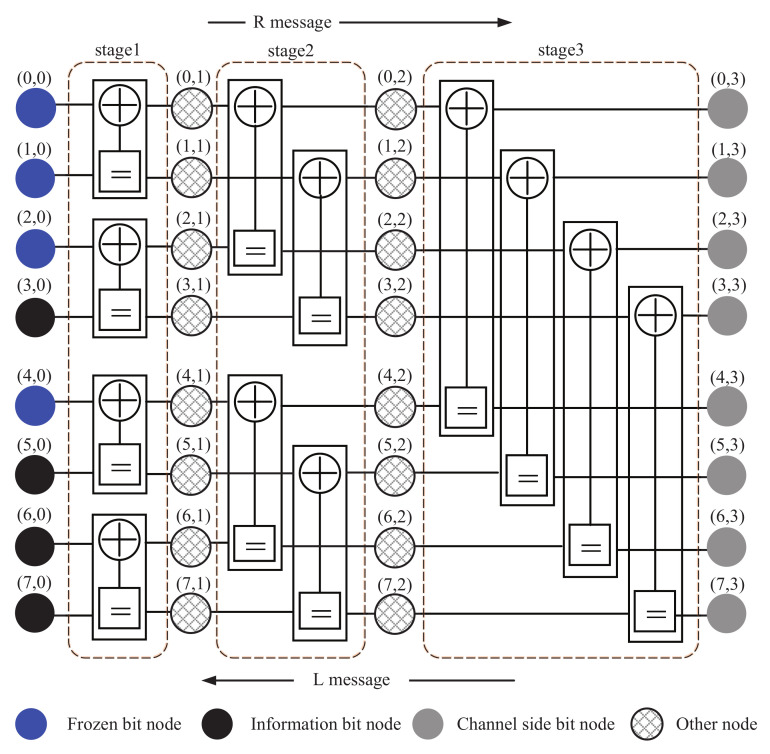
Decoding factor graph with *N* = 8.

The BP algorithm for polar codes is based on a DFG. A polar code with code length *N* is represented by an *n*-stage DFG. We use (*i*, *j*) to indicate the nodes of the DFG, where *i* indicates node index and *j* indicates column index. The leftmost nodes in the DFG mean *j* = 0, such as the blue and black nodes in the Figure 1. Similarly, the rightmost nodes in the DFG mean *j* = *n*, as shown in the grey node column.

**Figure 2:**
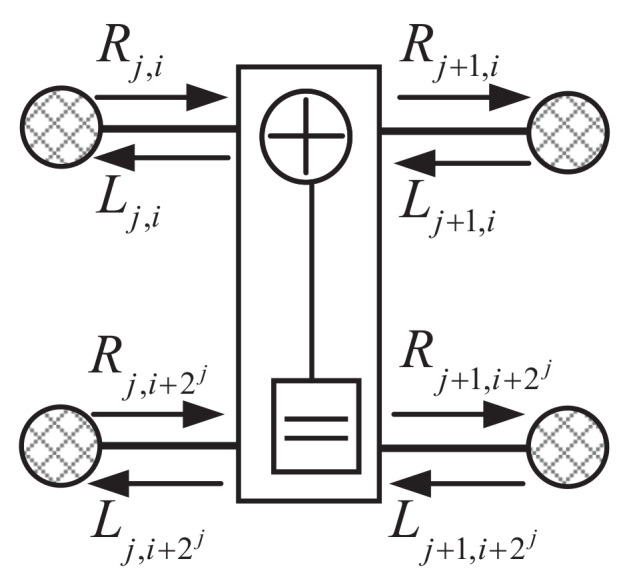
Fundamental PE.

The classic BP DFG is depicted in Figure 1. Each stage consists of N/2 processing elements (PE), where a fundamental PE is shown in Figure 2. Figure 1 consists of three stages and each stage has four PEs. One PE has four nodes and each node is associated with two types of messages, a right-to-left message $L_{j,i}$ and a left-to-right message $R_{j,i}$. $L_{j,i}$ and $R_{j,i}$ are in the form of LLR. The message propagation rules are as follows:

(5) $$\left\{\begin{array}{c} L_{j,i}=g\left(L_{j+1,i},R_{j,i+2^{j}}+L_{j+1,i+2^{j}}\right),\\ L_{j,i+2^{j}}=g\left(L_{j+1,i},R_{j,i}\right)+L_{j+1,i+2^{j}},\\ R_{j+1,i}=g\left(R_{j,i},L_{j,i+2^{j}}+R_{j+1,i+2^{j}}\right),\\ R_{j+1,i+2^{j}}=g\left(R_{j,i},L_{j+1,i}\right)+R_{j,i+2^{j}}. \end{array}\right.$$

where

(6) $$\begin{array}{c} g\left(x,y\right)=\alpha \cdot \mathrm{sign}\left(x\right)\cdot \mathrm{sign}\left(y\right)\cdot \min\left(\left|x|,|y\right|\right), \end{array}$$

where α = 0.9375 follows from the scaling factor used in [21]. $L_{j,i}$ and $R_{j,i}$ need to be initialized as follows

(7) $$L_{j,i}=\left\{\begin{array}{c} 0,\;\;\;\;i\neq n+1,\\ L_{n}^{\left(j\right)},\;\;\;\;\;\;i=n+1. \end{array}\right.$$

(8) $$R_{j,i}=\left\{\begin{array}{c} 0,\;\;\;\;j\in A,\\ +\infty ,\;\;\;\;\;\;i=1,\;\;j\in A^{C}, \end{array}\right.$$

where $L_{n}^{\left(j\right)}$ denotes the LLR of the *j*-th received bit. The +∞ in the first column of $R_{j,1}$ indicates the prior knowledge carried by the frozen bits.

The maximum number of iterations $I_{\max}$ is preset, and the decoding is terminated when the number of iterations is equal to $I_{\max}$ or when the CRC check is satisfied. The hard decisions of ${\hat{u}}_{i}$ and ${\hat{x}}_{i}$ are estimated as

(9) $$\begin{array}{c} {\hat{u}}_{i}=\left\{\begin{array}{c} 1,\;\;L_{j,1}+R_{j,1}<0,\\ 0,\;\;L_{j,1}+R_{j,1}\geq 0. \end{array}\right. \end{array}$$

(10) $$\begin{array}{c} {\hat{x}}_{i}=\left\{\begin{array}{c} 1,\;\;L_{j,n+1}+R_{j,n+1}<0,\\ 0,\;\;L_{j,n+1}+R_{j,n+1}\geq 0. \end{array}\right. \end{array}$$

### 2.3. BP Flipping Decoding Algorithm

The bit-flipping strategy is a feasible method with which to improve the performance of BP-based algorithms. Due to the parallelism of BP decoding, there may be more than one bit that could correct the error frame by flipping it. Not only can the real error bits correct the error frames, but there are still other bits that can correct the error frames in the process of the iterative computation of the DFG [18]. However, there exist more bits that provide no assistance in error frame correction, and the invalid flipping of these will cause much latency. Therefore, the study of the strategy used for locating the flipping bits which can effectively correct the error frames is essential.

**Figure 3:**
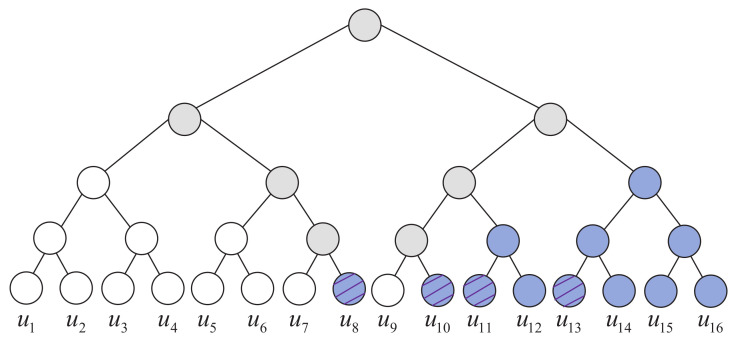
An example of Rate-1 with *P*(16, 8). White node means that all its leaf nodes are frozen bits, blue node means that all its leaf nodes are information bits, striped blue node denotes the first information bit of the blue node and grey node denotes that its leaf nodes include both information and frozen bits.

Initially, the CS is used to identify unreliable bits in the SC flipping decoder, which is composed of the first bit index of each Rate-1 node [15]. As shown in Figure 3, blue nodes are referred to as Rate-1 nodes because all the leaf nodes are information bits, white node means that all its leaf nodes are frozen bits and grey node denotes that its leaf nodes include both information and frozen bits. In Figure 3, CS = {8,10,11,13} is shown as striped blue nodes. It can be noticed that the size and elements of CS are fixed for a certain polar code, which means that CS is static in decoding. Because of this characteristic, no latency will be caused by FS construction. The BPF algorithm [15] employs CS in bit-flipping and the flipping operation is as follows

(11) $$R_{i,0}=\left\{\begin{array}{c} +\infty ,\;\;i\in CS\;\mathrm{and}\;{\hat{u}}_{i}=0,\\ -\infty ,\;\;i\in CS\;\mathrm{and}\;{\hat{u}}_{i}=1. \end{array}\right.$$

For the GBPF decoding [17,18] algorithm, the FS is constructed dynamically with the smallest LLR magnitude. A sorting network is required to select information bits to constitute the FS, $F_{1}^{\Gamma }$=$\left\{F_{1},F_{2},\ldots ,F_{\Gamma }\right\}$, where Γ denotes the length of the FS. Before the next-round of BP decoding attempts, the FS is generated by the smallest LLR magnitude and the bit-flipping operation is performed with the FS. The rule used to generate the FS is defined by

(12) $$FS\leftarrow j\in A\;\mathrm{and}\;\mathrm{smallest}\;|L_{n}^{\left(j\right)}|.$$

Specifically, the GBPF flipping operation can be written as

(13) $$R_{0,i}=\left\{\begin{array}{c} +\infty ,\;\;i\in FS\;\mathrm{and}\;{\hat{u}}_{i}=1,\\ -\infty ,\;\;i\in FS\;\mathrm{and}\;{\hat{u}}_{i}=0,\\ 0,\;\;\;\;i\in A/FS. \end{array}\right.$$

The oracle-assisted BP (OA-BP) decoder [18] knows which bit estimates make the frame mistakes after the original BP decoding and then re-decodes the incorrect frame by flipping the erroneous bit in turn into the correct value. The incorrect codeword set can be expressed as $A_{1}^{\tau }$ = $\left\{A_{1},A_{2},\ldots ,A_{\tau }\right\}$, where τ is the count of erroneous bits.

The differences between BPF algorithms are how to choose the flipping set. The BPF algorithm generates CS before decoding with Rate-1 nodes and stays static in decoding. The GBPF algorithm generates FS dynamically in decoding, which will lead to extra costs of sorting the LLR magnitude in generating FS. However, the GBPF algorithm also provides greater possibility of error-correction by setting larger sizes of FS. The generalized procedure for BPF-1 algorithm [15] and GBPF-1 algorithm [17] can be summarized as Algorithm 1.
**Algorithm 1** BP flipping algorithm. 1:**Input:** $y_{1}^{N}$, Γ, $\rho_{1}$2:**Output:** ${\hat{u}}_{1}^{N}$3:$\{{\hat{u}}_{1}^{N}\}\leftarrow$ BP $\left(y_{1}^{N}\right)$;4:**if** CRC(${\hat{u}}_{1}^{N}$) fail5:   **for** $\rho_{1}\leftarrow$ 1 to Γ **do**6:      $\{{\hat{u}}_{1}^{N}\}\leftarrow$ BP $\left(y_{1}^{N},\rho_{1}\right)$;7:      **if** CRC (${\hat{u}}_{1}^{N}$) succeed8:         **break**;9:      **end if**10:   **end for**11:**end if**12:**return ${\hat{u}}_{1}^{N}$**

**Figure 4:**
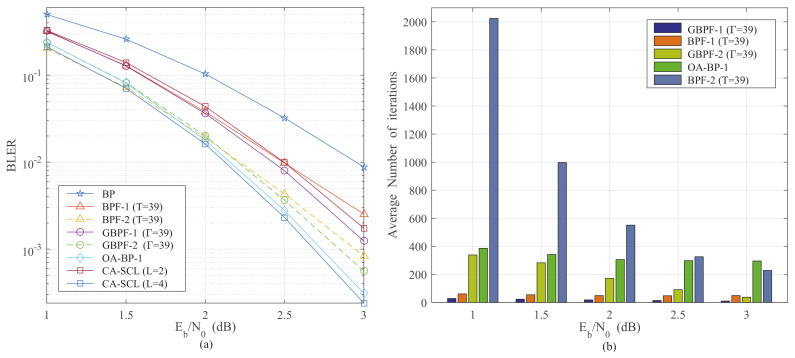
The BLER performance and the average number of iterations for the BP [10] (Hussami, N.; Korada, S.B.; Urbanke, R. 2009), BPF-1, BPF-2 [15] (Yu, Y.; Pan, Z.; Liu, N.; You, X. 2019), GBPF-1 [17] (Shen, Y.; Song, W.; Ren, Y.; Ji, H.; You, X.; Zhang, C. 2020), GBPF-2, OA-BP-1 [18] (Shen, Y.; Song, W.; Ji, H.; Ren, Y.; Ji, C.; You, X.; Zhang, C 2020) and CA-SCL [9] (Niu, K.; Chen, K. 2012) algorithm. (**a**) The BLER comparison of existing algorithms with *N* = 256. (**b**) The average number of iterations for the existing algorithms with *N* = 256.

The bits needing to be flipped by the BP flipping algorithms are listed in Table 1. The BPF algorithm has a significantly higher number of flipping bits than the GBPF algorithm. The BLER performance and the average number of iterations for the existing algorithms are shown in Figure 4. It can be seen that the BLER performance of the BPF-Ω and GBPF-Ω algorithms is competitive. However, the average number of iterations for the BPF and GBPF algorithms increases exponentially with the rise of Ω. The average number of iterations of the BPF-2 algorithm is more than two thousand at $E_{b}/N_{0}$ = 1. Therefore, we propose a step-flipping strategy to reduce the average number of iterations in Section 3.

## 3. The Proposed BPSF Algorithm

In this section, we first analyze the behavior of the CS. Then, the BPSF algorithm is proposed to reduce the average number of iterations. Finally, the threshold factor is applied to the BPSF algorithm to lower the BLER, and the pseudocode of the two-bit-flipping algorithm is presented.

### 3.1. Analysis of Critical Set

**Figure 5:**
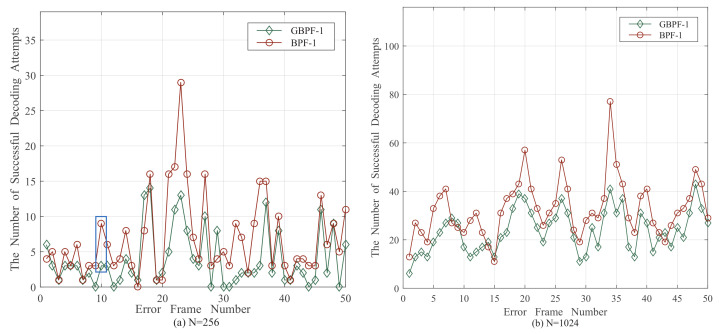
The number of successful BP flipping decoding attempts by the BPF-1 [15] (Yu, Y.; Pan, Z.; Liu, N.; You, X. 2019), GBPF-1 algorithms [17] (Shen, Y.; Song, W.; Ren, Y.; Ji, H.; You, X.; Zhang, C. 2020) at 2 dB. (**a**) The number of successful BP flipping decoding attempts with *N* = 256. (**b**) The number of successful BP flipping decoding attempts with *N* = 1024.

According to Section 2.3, the size of the critical set is determined by the Rate-1 nodes. For *N* = 256, the size of CS is *T* = 39. Similarly for *N* = 512 and *N* = 1024, *T* equals 60 and 116 respectively. There are many elements in CS that could correct the error frames by flipping them and one flipping means one attempt. The BPF-1 and GBPF-1 decoders are analyzed in Figure 5 when *N* = 256 and *N* = 1024. It illustrates the number of successful flipping attempts in the CS-T that could correct the error frames by flipping them at 2 dB.

As shown in Figure 5a, there are nine attempts of BPF-1 decoding and three attempts of GBPF-1 decoding that could correct the error. Frame 10 is marked with a blue rectangle, but the other decoding attempts are failed to correct this error frame. Those successful decoding attempts can be expressed as $r_{\Omega }^{M}=\left\{r_{\Omega }^{1},r_{\Omega }^{2},\cdots ,r_{\Omega }^{M}\right\}$, where Ω is the maximum bit-flipping order and *M* is the number of successful BP-flipping decoding attempts. When Ω = 1, *M* is less or equal to the number of BP-flipping decoding attempts ($C_{T}^{1}=39$). When Ω = 2, *M* is less or equal to the number of BP-flipping decoding attempts ($C_{T}^{1}+C_{T}^{2}=780$). The decoding attempts are similar when *N* = 1024, as shown in Figure 5b.

The distribution of *M* is listed in Table 2. In the BPF-1 and GBPF-1 decoders, 87.9% and 79.3% of the error frames can be corrected with *M* ≤ 9. It means that the number of successful decoding attempts is not larger than 9 for most error frames. Likewise, 79.1% and 78.3% of the error frames can be corrected with *M* ≤ 99 in the BPF-2 and GBPF-2 decoders. It can be observed that only a few BP-flipping decoding attempts can correct the error frames while the others are not helpful. In addition, the BP-flipping decoding attempts are different for each error frame. Thus, we propose a method to detect ineffective decoding attempts and step them to reduce the average number of decoding attempts.

### 3.2. Proposed BPSF Algorithm

A flipping set of FS-*T* can be expressed as $\varepsilon_{T}$ =$\left\{\omega_{1},\cdots ,\omega_{T}\right\}$ and $\omega_{q}$ (1≤q≤T) indicates the *q*-th flipping position. $\hat{u}{\left[\omega_{q}\right]}_{j}$ denotes the hard decision estimate of the bit $u_{j}$ after flipping the $\omega_{q}$-th bit. Let $P(\omega_{q})$ be the probability of $\omega_{q}$ correcting the trajectory of BP decoding, where

(14) $$\begin{array}{c} P\left(\omega_{q}\right)=\mathrm{Pr}\left(\hat{u}{\left[\omega_{q}\right]}_{1}^{N}=u_{1}^{N}|y\right) \end{array}$$

**Figure 6:**
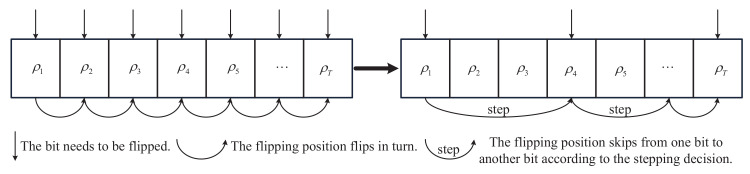
The flipping sequence of original BPF and BPF with stepping strategy.

Experiments show that only a few bits in the FS-*T* can correct the error frames, and flipping the rest will not facilitate successful decoding. Thus, this paper proposes a stepping scheme to step the flipping bits with low $P(\omega_{q})$. Let $L_{j,0}^{\left(\omega_{q}\right)}$ denote the LLR of the bit $u_{j}$ after flipping the $\omega_{q}$-th bit. The LLR magnitude can be intuitively used as a metric of the reliability [21]. If the $L_{\omega_{q},0}^{\left(\omega_{q}\right)}$ magnitude is smaller, the bit is considered as an unreliable bit and then the $P(\omega_{q})$ of the bit is deemed to be higher. Thus, if the $L_{\omega_{q},0}^{\left(\omega_{q}\right)}$ magnitude is smaller than the $L_{\omega_{q+1,0}}^{\left(\omega_{q}\right)}$ magnitude, $P(\omega_{q})$ is deemed to be higher than $P(\omega_{q+1})$ and there is no need to perform a useless flipping for the reliable $\omega_{q+1}$ bit. Let ρ = $\left\{\rho_{1},\rho_{2},\cdots ,\rho_{T}\right\}$ denote the index of FS. We assume one decoding trial of flipping $\omega_{\rho_{i}}$ has failed. Additionally, we obtain the new LLR of $L_{\omega_{\rho_{i}},0}^{\left(\omega_{\rho_{i}}\right)}$ and $L_{\omega_{\rho_{k},0}}^{\left(\omega_{\rho_{i}}\right)}$, i<k≤T. The stepping decision is as (15). Using the stepping decision, the flipping sequence of the original BPF is modified, as shown in Figure 6.

(15) $$\begin{array}{c} \rho_{k}\;is\;skipped,\;\mathrm{if}\;|L_{\omega_{\rho_{i}},0}^{\left(\omega_{\rho_{i}}\right)}\left|<\right|L_{\omega_{\rho_{k},0}}^{\left(\omega_{\rho_{i}}\right)}| \end{array}.$$

The original flipping strategy used in BPF [15] is to flip bits within CS-*T* in turn. We propose the use of the stepping decision in the BPSF algorithm to decide which bit can be skipped. The flipping operation in the proposed algorithm is as (16), which flips the left message $L_{j,n+1}$ of the rightmost nodes.

(16) $$\begin{array}{c} L_{j,n+1}^{}=\left\{\begin{array}{c} -\infty ,\;\mathrm{if}\;L_{n}^{\left(j\right)}\geq 0,\\ +\infty ,\;\mathrm{if}\;L_{n}^{\left(j\right)}<0. \end{array}\right. \end{array}$$

Similarly, inspired by the GBPF-Ω algorithm [17,18], we design a generalized bit step-flipping (GBPSF-Ω) algorithm. There are two differences between the GBPF-Ω and GBPSF-Ω. Firstly, GBPSF-Ω flips the left message of the rightmost nodes according to (16). Secondly, the stepping decision is used to step over bits, as shown in Figure 6, after the construction of FS.

### 3.3. Enhanced BPSF Algorithm

**Figure 7:**
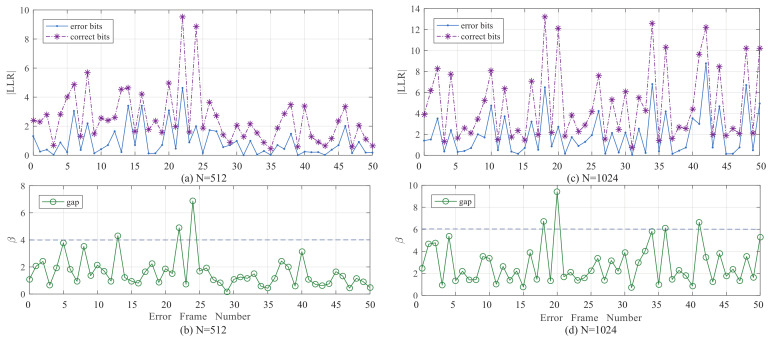
The LLR magnitude and the gap β between the error bits and the correct bits at 2 dB. (**a**) The LLR magnitude for *P*(512, 256). (**b**) The gap β between the error bits and the correct bits for *P*(512, 256). (**c**) The LLR magnitude for *P*(1024, 512). (**d**) The gap β between the error bits and the correct bits for *P*(1024, 512).

The BPSF-Ω algorithm can significantly decrease the average number of iterations, but some flipping bits that could correct the frame in FS are skipped when their LLR magnitude is small, which will cause performance degradation. In Figure 7a,c, the “blue line” denotes the LLR magnitude of $\omega_{j}$ (1≤j≤M), which cannot correct the error frame after one BP decoding attempt. The ”Purple line” denotes the LLR magnitude of $\omega_{k}$ (j<k≤M), which is the first bit after $\omega_{k}$ that can correct the error frame. The LLR magnitude gap between $\omega_{k}$ and $\omega_{j}$, called β, is shown in Figure 7b,d. It indicates that $\omega_{k}$ can still be an unreliable bit even if it satisfies (15).

Table 3 illustrates that the β value mostly concentrates on the range [1 2] and [0 2]. Using the β, the stepping decision of (15) is modified to (17) to skip the unreliable bits more accurately. Then, the enhanced BPSF-Ω algorithm (EBPSF-Ω) and the enhanced GBPSF-Ω algorithm (EGBPSF-Ω) with the threshold β are developed, whose stepping decision is the same as (17). β can be determined by a Monte-Carlo simulation. We detail the EBPSF-2 algorithm in Algorithm 2, and the EBPSF-Ω algorithm is also similar to it when Ω>2.

(17) $$\begin{array}{c} \rho_{k}\;is\;skipped,\;\mathrm{if}\;|L_{\omega_{\rho_{i}},0}^{\left(\omega_{\rho_{i}}\right)}\left|-\beta <\right|L_{\omega_{\rho_{k}},0}^{\left(\omega_{\rho_{i}}\right)}| \end{array}.$$

**Algorithm 2** EBPSF-2 decoding.
 1:**Input:** $y_{1}^{N}$, $\omega_{\rho }$, β, *T*2:**Output:** ${\hat{u}}_{1}^{N}$3:$\{{\hat{u}}_{1}^{N}\}\leftarrow$ BP $\left(y_{1}^{N}\right)$;4:**if** CRC(${\hat{u}}_{1}^{N}$) fail5:   **while** $\rho_{1}<T$ **do**6:      flip ($L_{n}^{\left(\omega_{\rho_{1}}\right)}$) based (16);7:      $\{{\hat{u}}_{1}^{N}\}\leftarrow$ BP $\left(y_{1}^{N}\right)$;8:      **if** CRC (${\hat{u}}_{1}^{N}$) fail9:         update $\rho_{1}$ according to (17);10:      **else**11:         **break**;12:       **end if**13:    **end while**14:    **if** CRC (${\hat{u}}_{1}^{N}$) fail15:       $\rho_{2}=\rho_{1}+1$;16:       **while** $\rho_{1}<T$ **do**17:          **while** $\rho_{2}<T$ **do**18:             flip ($L_{n}^{\left(\omega_{\rho_{1}}\right)}$,$L_{n}^{\left(\omega_{\rho_{2}}\right)}$) based (16);19:             $\{{\hat{u}}_{1}^{N}\}\leftarrow$ BP $\left(y_{1}^{N}\right)$;20:             **if** CRC (${\hat{u}}_{1}^{N}$) fail21:                update $\rho_{1}$ according to (17);22:                update $\rho_{2}$ according to (17);23:             **else**24:                **break**;25:             **end if**26:          **end while**27:          **if** CRC (${\hat{u}}_{1}^{N}$) succeed28:             **break**;29:          **end if**30:       **end while**31:    **end if**32: **end if**33: **return** ${\hat{u}}_{1}^{N}$


## 4. Numerical Results

In this section, we compare the proposed step-flipping algorithm and the existing flipping algorithm in terms of β, the average number of iterations, and the BLER with different code lengths. Simulations are performed with additive white Gaussian noise (AWGN) channel and binary-phase shift keying (BPSK) modulation. The additional simulation parameters are listed in Table 4. The simulations for the (256, 128), (512, 256), and (1024, 512) polar codes are concatenated with 24-bit CRC and for (64, 32) polar codes with 11-bit CRC. The *m* CRC bits are attached to the information block, where *m* is the CRC remainder length, and all the *K* bits are sent into the error-correcting encoders.

### 4.1. Analysis of the Threshold β

**Figure 8:**
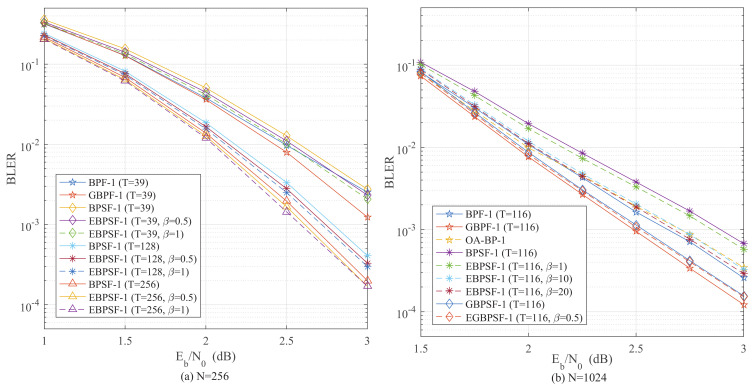
The BLER of BPF-1 [15] (Yu, Y.; Pan, Z.; Liu, N.; You, X. 2019), GBPF-1 [17] (Shen, Y.; Song, W.; Ren, Y.; Ji, H.; You, X.; Zhang, C. 2020), OA-BP-1 [18] (Shen, Y.; Song, W.; Ji, H.; Ren, Y.; Ji, C.; You, X.; Zhang, C 2020), BPSF-1, EBPSF-1, GBPSF-1 and EGBPSF-1 with different threshold β. (**a**) The BLER comparison of different algorithms for *P*(256, 128). (**b**) The BLER comparison of different algorithms for *P*(1024, 512).

To verify the effectiveness of the threshold β, the BLERs of the BPSF-1, EBPSF-1, GBPSF-1 and EGBPSF-1 algorithms with different threshold β are compared in Figure 8. Additionally, the GBPF-1 and BPF-1 algorithms are provided as references.

With the assistance of β, the EBPSF-1 algorithm outperforms the BPSF-1 algorithm in BLER under the same parameters for both N=256 in Figure 8a and N=1024 in Figure 8b. For β = 0.5 and β = 1 when N=256 and *T* = 128, the EBPSF-1 algorithm achieves 0.09 dB and 0.13 dB gain with the BPSF-1 algorithm at BLER = $1\times 10^{-2}$, respectively. Furthermore, for β = 1 and β = 10 when N=1024 and *T* = 116, the EBPSF-1 algorithm obtain the gain of 0.04 dB and 0.23 dB for BLER = $10^{-3}$ compared with the BPSF-1 algorithm. Therefore, the EBPSF-1 algorithm has more accuracy in locating the reliable bits among the CS-*T*. With the increase of β, the BLER performance of the EBPSF algorithm is continuously optimized, but the average number of iterations also increases. Therefore choosing a proper β is essential to optimizing the BLER performance and can lead to a negligible increase in the average number of iterations.

### 4.2. Analysis of the Average Number of Iterations

**Figure 9:**
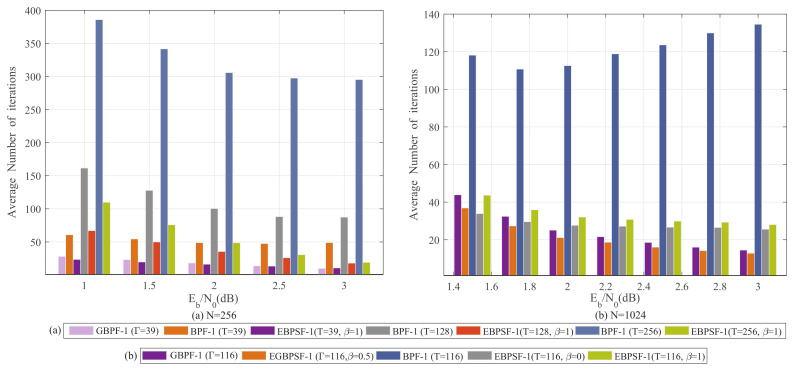
The average number of iterations for GBPF-1 [17] (Shen, Y.; Song, W.; Ren, Y.; Ji, H.; You, X.; Zhang, C. 2020), BPF-1 [15] (Yu, Y.; Pan, Z.; Liu, N.; You, X. 2019), EBPSF-1 and EGBPSF-1. (**a**) The average number of iterations for different algorithms with *N* = 256. (**b**) The average number of iterations for different algorithms with *N* = 1024.

**Figure 10:**
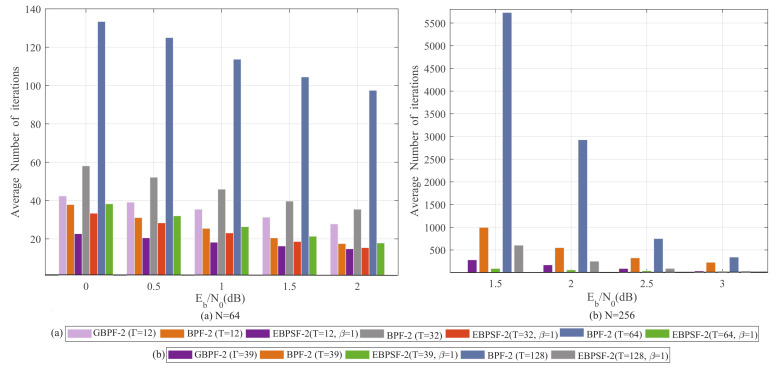
The average number of iterations for GBPF-2 [18] (Shen, Y.; Song, W.; Ji, H.; Ren, Y.; Ji, C.; You, X.; Zhang, C 2020), BPF-2 [15] (Yu, Y.; Pan, Z.; Liu, N.; You, X. 2019) and EBPSF-2. (**a**) The average number of iterations for different algorithms with *N* = 64. (**b**) The average number of iterations for different algorithms with *N* = 256.

The stepping strategy is used to skip some bits in FS. Thus, the number of flippings is smaller than the original flipping algorithm without the stepping strategy. To verify this point, some simulations are performed. Figure 9 and Figure 10 compare the average number of iterations among the BPF-Ω, GBPF-Ω, EBPSF-Ω and EGBPSF-Ω algorithms. The average number of iterations for the EBPSF-Ω algorithm is lower than the BPF-Ω algorithm, while the EGBPSF-Ω algorithm is lower than that of the GBPF-Ω algorithm. That’s because the step-flipping strategy applied in the EBPSF-Ω and EGBPSF-Ω algorithms reduces flipping attempts by skipping the reliable flipping bits.

It can be observed from Figure 9 and Figure 10 that there is a significant decrease in the average number of iterations when applying the step-flipping strategy. At high $E_{b}/N_{0}$, the EBPSF-Ω (*T* = 256) algorithm is close to the BPF-Ω (*T* = 39) algorithm in the average number of iterations. For *T* = 39, the EBPSF-1 (β = 1) algorithm reduces the average number of iterations by 14.1% and 62.2% at 1.5 dB compared with the GBPF-1 and BPF-1 algorithms, respectively. In comparison with the GBPF-1 algorithms, the EGBPSF-1 (Γ = 116, β = 0) algorithm reduces the average number of iterations by 16.28% at 1.5 dB.

Similarly, for the two-bit flipping, the average number of iterations is shown in Figure 10. Under the same *T*, the average number of iterations for the EBPSF-2 algorithm is significantly lower than that for the BPF-2 and GBPF-2 algorithms. In the case of 0 dB and *T* = 12, the EBPSF-2 algorithm is inferior to BPF-2 and GBPF-2 algorithms by 40.54% and 47.62%, as shown in Figure 10a, respectively. In Figure 10b, with *T* = 128 and *T* = 256, the EBPSF-2 algorithm reduces the average number of iterations by 77.4% and 95.9% at 1.5 dB against the BPF-2 algorithm, respectively. Consequently, the step-flipping strategy that we propose for the BP one-bit flipping and multi-bit flipping algorithms is effective in reducing the average number of iterations.

### 4.3. Analysis of the BLER Performance

**Figure 11:**
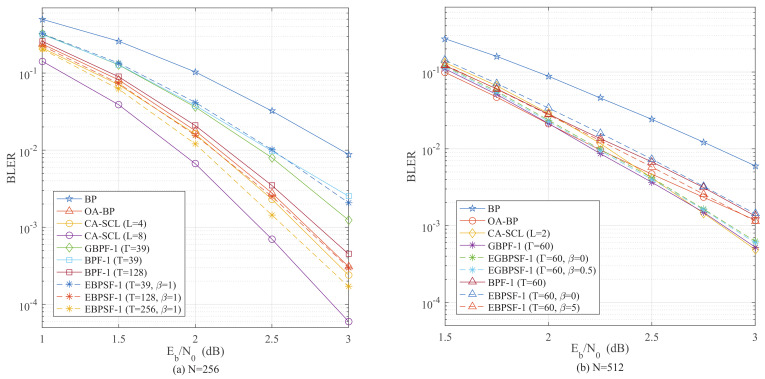
The BLER comparison of BP [10] (Hussami, N.; Korada, S.B.; Urbanke, R. 2009), OA-BP-1 [18] (Shen, Y.; Song, W.; Ji, H.; Ren, Y.; Ji, C.; You, X.; Zhang, C 2020), CA-SCL [9] (Niu, K.; Chen, K. 2012), GBPF-1 [17] (Shen, Y.; Song, W.; Ren, Y.; Ji, H.; You, X.; Zhang, C. 2020), BPF-1 [15] (Yu, Y.; Pan, Z.; Liu, N.; You, X. 2019), EGBPSF-1 and EBPSF-1. (**a**) The BLER comparison of different algorithms for *P*(256, 128). (**b**) The BLER comparison of different algorithms for *P*(512, 256).

**Figure 12:**
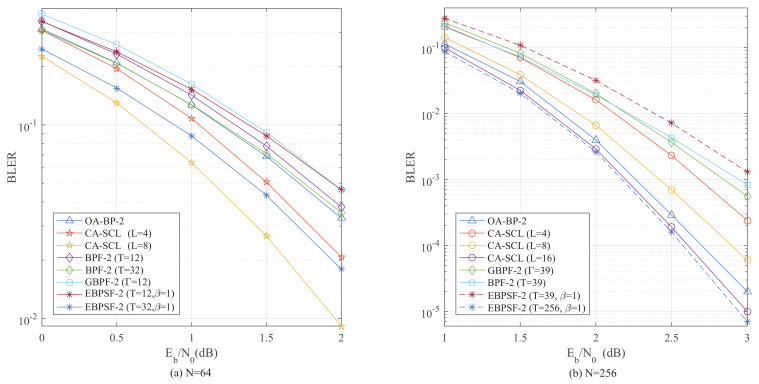
The BLER comparison of OA-BP-2 [18] (Shen, Y.; Song, W.; Ji, H.; Ren, Y.; Ji, C.; You, X.; Zhang, C 2020), CA-SCL [9] (Niu, K.; Chen, K. 2012), GBPF-2 [18] (Shen, Y.; Song, W.; Ji, H.; Ren, Y.; Ji, C.; You, X.; Zhang, C 2020), BPF-2 [15] (Yu, Y.; Pan, Z.; Liu, N.; You, X. 2019) and EBPSF-2. (**a**) The BLER comparison of different algorithms for *P*(64, 32). (**b**) The BLER comparison of different algorithms for *P*(256, 128).

Using the stepping strategy, some bits in FS are skipped during the procedure of flipping with negligible performance loss. The BLER performance of the EBPSF-Ω and EGBPSF-Ω algorithms is depicted in Figure 11 and Figure 12. It can be observed that the algorithms which apply the proposed step-flipping strategy are better in terms of the BLER performance. Unlike the BPF-Ω and GBPF-Ω algorithms, which flip the right information of the leftmost nodes in the DFG, we flip the left information of the rightmost nodes by (16). In contrast to the CA-SCL decoding, the EBPSF-Ω and EGBPSF-Ω algorithms can achieve comparable decoding performance.

When *T* = 39, Figure 11a indicates that the EBPSF-1 (β = 1) algorithm performs similarly to the BLER of GBPF-1 algorithm. Additionally, the EBPSF-1 algorithm has 0.23 dB gain at BLER = $4\times 10^{-3}$ against the OA-BP decoder when *T* = 256. The BPF-Ω and GBPF-Ω algorithms flip the right information of the leftmost nodes in the DFG. However, the proposed algorithm flips the left information of the rightmost nodes, and the EBPSF algorithm obtains a gain over the OA-BP decoder. With the same parameters, the EBPSF-1 (*T* = 256) outperforms the CA-SCL (*L* = 4) decoder by 0.13 dB when BLER = $1\times 10^{-2}$. The one-bit flipping BLER performance for *N* = 512 is depicted in Figure 11b. When *T* = 60 and β = 0.5, the EBPSF-1 algorithm for BLER compared to the BPF-1 algorithm shows a gain of 0.09 dB at BLER = $2\times 10^{-3}$. At *T* = 60, the performance of the EGBPSF-1 (Γ = 60, β = 0.5) algorithm is approaching the BLER of the CA-SCL (*L* = 2) decoder.

The two-bit flipping performance for *N* = 64 is illustrated in Figure 12a. With *T* = 32, the EBPSF-2 (β = 1) algorithm has 0.47 dB, 0.53 dB and 0.41 dB gain compared with the BPF-2, GBPF-2 and OA-BP-2 algorithms at BLER = $5\times 10^{-2}$, respectively. Furthermore, the EBPSF-2 (*T* = 32, β = 1) algorithm achieves the BLER performance between CA-SCL (*L* = 4) and (*L* = 8). Figure 12b presents the two-bit flipping performance for *N* = 256. The EBPSF-2 (β = 1) algorithm, when *T* = 256, shows an improvement of 0.21 dB compared with the OA-BP-2 algorithm at BLER = $5\times 10^{-4}$. The EBPSF-2 (*T* = 256, β = 1) algorithm outperforms the CA-SCL (*L* = 16) decoder by 0.02 dB at BLER = $1\times 10^{-3}$.

## 5. Conclusions

To reduce the average number of iterations in the BP flipping algorithm, this paper proposes a BP flipping algorithm with the stepping strategy. To narrow the search space, the reliable bits are skipped by the stepping strategy to improve the accuracy of flipping bits. The magnitude of LLRs are used to determine whether the bits are reliable or not, skip over the reliable bits in the FS, and flip the unreliable bits to reduce the average number of iterations. Furthermore, to make the algorithm more robust, we propose the threshold β to reduce the BLER. Simulation results show that the proposed algorithm with one-bit flipping and two-bit flipping can achieve BLER for CA-SCL decoder with list size 4 and 8, separately. Compared with the BPF decoder and the GBPF decoder, the EBPSF-Ω significantly reduces the average number of iterations. 

## Figures and Tables

**Table 1:** Statistics for the flipping bits.

Algorithm	One-Bit-Flipping Digits	Two-Bit-Flipping Digits
BPF [15]	2×T	$2^{2}\times C_{T}^{2}$
GBPF [17,18]	Γ	$C_{\Gamma }^{2}$
OA-BP [18]	τ	$C_{\tau }^{2}$

**Table 2:** Statistics for the number of successful BP-flipping decoding attempts in 10,000 error frames at *N* = 256, $E_{b}/N_{0}$ = 2 dB.

***M***	*M* ≤ 9	10 ≤ *M* ≤ 19	20 ≤ *M* ≤ 39
BPF-1	87.9%	11.7%	0.4%
GBPF-1	79.3%	17.6%	3.1%
***M***	***M* ≤ 99**	**100 ≤ *M* ≤ 399**	**400 ≤ *M* ≤ 780**
BPF-2	79.1%	17.7%	3.2%
GBPF-2	78.3%	18.1%	3.6%

**Table 3:** Statistics for the distribution of β when *N* = 512 and *N* = 1024.

*N* = 512	0–1	1–2	2–4	>4
The distribution of β	26.9%	46.9%	19.7%	6.2%
***N* = 1024**	**0–2**	**2–4**	**4–6**	**>6**
The distribution of β	48.1%	31.9%	11.7%	8.3%

**Table 4:** Simulation Parameters.

Name	Parameters
Polar Codes	(64, 32), (256, 128), (512, 256) and (1024, 512)
Signal Channel	AWGN
Modulation Method	BPSK
Construction Method	GA
Frame Number	500,000
Rate	1/2
Maximum Iterations	40
CRC Generator Polynomial [22]	g(x) = $x^{11}$+$x^{10}$+$x^{9}$+$x^{5}$+1
	g(x) = $x^{24}$+$x^{23}$+$x^{18}$+$x^{17}$+$x^{14}$+$x^{11}$+$x^{10}$+$x^{7}$+$x^{6}$+$x^{5}$+$x^{4}$+$x^{3}$+$x^{1}$+1
Horizontal Coordinate	$E_{b}/N_{0}$
Design SNR of Construction	1 dB
Efficient Information Bits	*K* - *m*

## Data Availability

Not applicable.

## Abbreviations

The following abbreviations are used in this manuscript:

BP	Belief propagation
BLER	Block error rate
5G	Fifth generation
SC	Successive cancellation
FS	Flip set
CS	Critical set
LLR	Log-likelihood ratio
CRC	Cyclic redundancy check
PE	Processing elements
DFG	Decoding factor graph
BPSK	Binary phase shift keying
AWGN	Additive white gaussian noise

## References

[1] Arıkan E. Channel polarization: A method for constructing capacity-achieving codes. Proceedings of the 2008 IEEE International Symposium on Information Theory.

[2] (2016). Final Report of 3GPP TSG RAN WG1 #87 v1.0.0. https://www.3gpp.org/ftp/tsg_ran/WG1_RL1/TSGR1_87/Report/Final_Minutes_report_RAN1%2387_v100.zip.

[3] Gallager R. (1962). Low-density parity-check codes. IRE Tran. Inf. Theory..

[4] Balatsoukas-Stimming A., Giard P., Burg A. Comparison of Polar Decoders with Existing Low-Density Parity-Check and Turbo Decoders. Proceedings of the 2017 IEEE Wireless Communications and Networking Conference Workshops (WCNCW).

[5] Arıkan E. (2009). Channel Polarization: A method for constructing capacity-achieving codes for symmetric binary-input memoryless channels. IEEE Trans. Inf. Theory..

[6] Tal I., Vardy A. (2015). List Decoding of Polar Codes. IEEE Trans. Inf. Theory..

[7] Zhang Z., Qin K., Zhang L., Chen G.T. Progressive bit-flipping decoding of polar codes over layered critical sets. Proceedings of the IEEE Global Communications Conference (GLOBECOM).

[8] Chandesris L., Savin V., Declercq D. (2018). Dynamic-SCFlip decoding of polar codes. IEEE Trans. Commu..

[9] Niu K., Chen K. (2012). CRC-aided decoding of polar codes. IEEE Commu. Lett..

[10] Hussami N., Korada S.B., Urbanke R. Performance of polar codes for channel and source coding. Proceedings of the 2009 IEEE International Symposium on Information Theory.

[11] Elkelesh A., Ebada M., Cammerer S., ten Brink S. (2018). Belief Propagation List Decoding of Polar Codes. IEEE Commun. Lett..

[12] Elkelesh A., Ebada M., Cammerer S., ten Brink S. Belief propagation decoding of polar codes on permuted factor graphs. Proceedings of the 2018 IEEE Wireless Communications and Networking Conference (WCNC).

[13] Doan N., Hashemi S.A., Mondelli M., Gross W.J. On the Decoding of Polar Codes on Permuted Factor Graphs. Proceedings of the 2018 IEEE Global Communications Conference (GLOBECOM).

[14] Elkelesh A., Cammerer S., Ebada M., ten Brink S. Mitigating clipping effects on error floors under belief propagation decoding of polar codes. Proceedings of the 2017 International Symposium on Wireless Communication Systems (ISWCS).

[15] Yu Y., Pan Z., Liu N., You X. (2019). Belief Propagation Bit-Flip Decoder for Polar Codes. IEEE Access.

[16] Zhang J., Wang M. (2020). Belief Propagation Decoder With Multiple Bit-Flipping Sets and Stopping Criteria for Polar Codes. IEEE Access.

[17] Shen Y., Song W., Ren Y., Ji H., You X., Zhang C. (2020). Enhanced Belief Propagation Decoder for 5G Polar Codes With Bit-Flipping. IEEE Trans. Circuits Syst. II Exp. Briefs.

[18] Shen Y., Song W., Ji H., Ren Y., Ji C., You X., Zhang C. (2020). Improved Belief Propagation Polar Decoders With Bit-Flipping Algorithms. IEEE Trans. Commun..

[19] Yang Y., Yin C., Jan Q., Hu Y., Pan Z., Liu N., You X. Noise-Aided Belief Propagation List Bit-Flip Decoder for Polar Codes. Proceedings of the 2020 International Conference on Wireless Communications and Signal Processing (WCSP).

[20] Tal I., Vardy A. (2013). How to construct polar codes. IEEE Trans. Inf. Theory..

[21] Yuan B., Parhi K.K. (2014). Early Stopping Criteria for Energy-Efficient Low-Latency Belief-Propagation Polar Code Decoders. IEEE Trans. Signal Process..

[22] (2021). Technical Specification Group Radio Access Network. https://www.3gpp.org/ftp/Specs/archive/38_series/38.212/38212-g60.zip.

